# Behavioural and social drivers of routine childhood immunization in selected low coverage areas in the Philippines

**DOI:** 10.1186/s41256-025-00447-5

**Published:** 2025-09-29

**Authors:** Soledad Natalia Dalisay, Madilene Landicho, Maria Margarita Lota, Yoshiki Fujimori, Paulyn Jean Acacio-Claro, Evalyn Roxas, Alvin Abeleda, Jan Zarlyn Rosuello, Micaella Dato, Florian Vogt, Margaret Danchin, Vicente Belizario, Jessica Kaufman

**Affiliations:** 1 Department of Anthropology, College of Social Sciences and Philosophy, University of the Philippines, Diliman, Quezon City, Philippines; 2 Third World Studies Center, College of Social Sciences and Philosophy, University of the Philippines Diliman, Quezon City, Philippines; 3 College of Public Health, University of the Philippines Manila, Manila, Philippines; 4 Faculty of Social Sciences, Tampere University, Tampere, Finland; 5 The Kirby Institute, University of New South Wales, Sydney, Australia; 6 National Centre for Epidemiology and Population Health, Australian National University, Canberra, Australia; 7 Vaccine Uptake Group, Murdoch Children’s Research Institute, Melbourne, Australia; 8 Department of Paediatrics, University of Melbourne, Parkville, Australia

**Keywords:** Routine childhood vaccination, Behavioural and social drivers, Vaccine hesitancy, Philippines

## Abstract

**Background:**

Routine childhood vaccination coverage under the National Immunization Program of the Philippines is well below the target of 95% with a high number of zero-dose children. Declining immunization rates led to outbreaks of vaccine-preventable diseases such as measles, polio, and pertussis. This study aimed to identify the factors affecting childhood vaccine uptake by exploring the perspectives of community members, program managers, and coordinators.

**Methods:**

Three regions with low vaccine coverage in the Philippines were selected as study sites. We conducted focus groups with adult caregivers of vaccinated and unvaccinated children aged 0–11 years recruited by *barangay* (community) health workers. Key informant interviews were also conducted with immunization program managers and coordinators from different administrative levels. Focus group and interview guides were informed by the World Health Organization’s Behavioural and Social Drivers (BeSD) of Vaccination framework. Transcripts were analysed by themes and deductive axial coding was used to categorize themes into BeSD domains and socioecological levels.

**Results:**

Twelve focus groups (n = 143) and 57 key informant interviews were done. Various behavioural and social drivers of vaccination present at different levels of the socioecological model affect vaccine decisions both positively and negatively. Under the ‘Thinking and feeling’ domain of the BeSD, at the intrapersonal level, the perception of benefits and negative side effects of routine vaccines were clear drivers of vaccination. In the ‘Social processes’ domain, factors at multiple socioecological levels such as the influence of family, barangay health workers, and community leaders were identified. Practical issues such as the availability of vaccines and accessibility of vaccination sites remain a barrier to vaccination.

**Conclusions:**

Availability of routine vaccines and accessibility to vaccination sites are major challenges in the Philippines. Acceptability of routine vaccines continue to be affected by previous controversies around the Dengue vaccine and the recent COVID-19 pandemic. Findings suggest that enhancing advocacy for immunization through continuing communication training for health care workers on health promotion and education regarding vaccination may contribute to increased vaccine uptake. Integration of immunization with other population-based health programs could be explored.

**Supplementary Information:**

The online version contains supplementary material available at 10.1186/s41256-025-00447-5.

## Introduction

Routine childhood vaccination is a vital public health strategy that reduces disease morbidity and mortality and is associated with growth, cognition, and schooling outcomes for children [1]. However, global immunization rates declined in 2020 and 2021, due to the multifactorial impacts of the COVID-19 pandemic [2, 3]. In 2021, there were 18.2 million “zero-dose” children, or children who have received no vaccines—an increase of 5 million annually since 2019 [4]. While some recovery has been noted in 2022 and 2023 [5], millions of children are still being left behind, particularly in low- and middle-income countries like the Philippines [6].

In the Philippines, vaccines for children under the age of 5 years are provided free of charge through the National Immunization Program (NIP), formerly known as the Expanded Programme on Immunization. The country comprises 18 regions, which are subdivided into provinces, highly urbanized cities and municipalities, and barangays or villages. Immunization service delivery is managed by the central office of the Department of Health (DOH) Disease Prevention and Control Bureau (DPCB) with program managers at regional levels that provide technical and logistic support. Program coordinators at the provincial and municipal levels oversee the implementation of the program at the *barangay* levels. Coverage for routine childhood immunization in the Philippines has been well below the target 95% vaccination rate since 2014 [5]. Low coverage has led to outbreaks of vaccine-preventable diseases such as polio in 2019–2020, measles in 2016–2019, and pertussis in 2024 [7–9]. The Philippines had the fifth highest number of zero-dose children globally in 2022 [10].

One potential factor contributing to low coverage in the Philippines is distrust in vaccines due to the *Dengvaxia* controversy in 2017, when the new dengue vaccine allegedly caused fourteen deaths in vaccinated children before the vaccine program was stopped [11]. The controversy fuelled the distrust not only in *Dengvaxia* but in vaccines in general [12]. Vaccine confidence was also affected by the COVID-19 pandemic, with UNICEF reporting in 2023 that vaccine confidence in the Philippines dropped by 25% from 2020 to 2022 [13]. The COVID-19 pandemic also hindered the implementation of many healthcare services and programs including the NIP [14]. People were hesitant to avail of public health services in their local health centres due to fear of exposure to the COVID-19 virus and infection [15]. Moreover, health resources, which were already limited to start with, were diverted to COVID-19 response measures, exacerbating existing accessibility and availability issues [16]. There is limited evidence assessing parent-level factors affecting childhood vaccination uptake from the post-pandemic period, when coverage has dropped significantly [17, 18]. Specific data on the drivers of vaccine uptake in low coverage areas from the perspective of both those seeking and those facilitating vaccination is needed to inform tailored interventions. This study aimed to identify the factors affecting childhood vaccine uptake by exploring the perspectives of community members, program managers, and coordinators.

## Methods

### Study design

In this qualitative study, we applied a constructivist approach, conducting focus group discussions (FGDs) with primary caregivers and key informant interviews (KIIs) with immunization program managers and coordinators to gather data on their experiences and perspectives on routine childhood vaccination. Data collection and analysis were informed by the World Health Organization’s (WHO) Behavioural and Social Drivers (BeSD) of Vaccination framework [20] and the Socioecological Model (SEM) [21]. The BeSD conceptualises barriers and facilitators of vaccine uptake according to four domains: thinking and feeling about vaccines, social processes, motivational factors, and practical issues. The SEM considers the levels of influence affecting vaccine uptake, from the intrapersonal outwards to the policy level. This study is presented using the Consolidated Criteria for Reporting Qualitative (COREQ) research [19].

Study sites with low routine vaccination coverage were identified n consultation with the DOH Public Health Operations Center (PHOC). Within the selected regions (Regions IV-B, V, and VIII), two provinces per region, two municipalities per province, and two barangays per municipality were selected in consultation with the DOH Centers for Health Development of the Region and the respective local health authorities. In 2021, the percentage of fully-immunized children in the study provinces ranged from 50 to 58% [22], well below the national target of 95% coverage [5].

### Participant recruitment

**Key informant interviews (KIIs).** KIIs were conducted with program managers from the different levels of the DOH and coordinators from local government units at the provincial and municipal levels. Eligible participants were directly involved in the NIP either as implementer or as a decision maker, such as the head of the office or a specific program or activity. Exclusion criterion was the unavailability of the key informant (KI) during the study period. KIs were identified through purposive methods and were invited by a member of the study team to take part in an individual or group interview. Interviews were primarily conducted face to face, with a few conducted online due to KI scheduling constraints. Participants provided written informed consent prior to participation.

**Focus group discussions (FGDs).** Inclusion criteria for the FGDs included primary caregivers in each study site with children aged 0–11 years who were vaccinated or unvaccinated. Caregivers were purposively sampled to ensure that participants of various age groups were included. Caregivers were recruited through the cooperation of Barangay Health Workers (BHWs). Caregivers included BHWs in some locations as well. Exclusion criteria included primary caregivers who were not available during the scheduled FGD and those who were only visiting the study site but were not permanent residents. Potential participants were invited to attend a meeting in a central location in the barangay during the day, where they could take part in a survey (described elsewhere) and/or an FGD. When people arrived, the study team greeted them, described the study, assessed their eligibility, and provided information and consent materials. FGD participants provided written informed consent. Approximately 8–15 participants for each FGD were recruited, which aligns with the range of participants recommended in the methodological literature [23, 24].

### Data collection

**Key informant interviews (KIIs). **The interview guide questions were based on the BeSD framework and the SEM. They covered the KI’s position in the office and the mandate of the office, their duties and responsibilities in relation to the NIP, views, thoughts and experiences with the NIP including vaccine uptake and hesitancy, perceived barriers and enablers of the NIP, training and coordination mechanisms and, their recommendations for improved uptake of the NIP [see Additional file 1]. The interview guide was translated into Filipino and back translated by native speakers. Interviews were done individually or in a group setting in Filipino and/or English. Each interview lasted for about 60 min.

**Focus group discussion (FGD). **The FGD guide was based on the BeSD tools [20] and was translated and back translated. It was pretested with community members and edited before use. The guide included four sections covering personal information of the participants, knowledge of the process of vaccination, reasons for vaccination or non-vaccination, and their recommendations for more effective health promotion strategies using a sample promotional audio-visual material used during the DOH's Supplemental Immunization Activity [see Additional file 2]. Each FGD lasted 60–90 min. A staff member from the Rural Health Unit (RHU) was present during the FGDs to translate the questions and responses from the participants to the local language, whenever necessary.

All KIIs and FGDs were facilitated by a member of the research team with qualitative research expertise (SND, female, PhD, medical anthropology; ML, female, MA, anthropology), with other researchers present to take notes and probe responses. KIIs and FGDs were audio recorded and transcribed by a member of the research team, supplemented by contemporaneous notes. Participants were given non-monetary tokens of gratitude for their participation in the study.

### Data analysis

Transcriptions were done verbatim and translated into English by a member of the research team. Another member back translated the English transcripts. Transcripts were analysed in Microsoft Word by SND and ML using standard methods of inductive and deductive thematic analysis [25]. First, potential themes were inductively generated based on the domains of the BeSD and the SEM. Both SND and ML familiarized themselves first with the data gathered and documented their reflections and insights on the raw data. Initial codes were generated which formed the basis of the potential themes and patterns. Then SND, ML and JK (female, PhD, public health) performed deductive axial coding to categorize the themes according to the BeSD and SEM conceptual frameworks. This two-step process has been applied previously to enable mapping of data-driven themes to potential strategies [26]. The data revealed emergent themes that were originally not identified, hence, the use of both deductive and inductive coding. The final themes were vetted by the rest of the research team members. Participant quotations are identified by their location (Region IV-B, Region V, Region VIII) and whether they were a participant in an FGD or KII. KII participants from the central government are identified by their department, rather than region (e.g., “DPCB KII”).

Trustworthiness of the research was ensured using the four pillars of trustworthiness in qualitative research, namely, credibility, dependability, confirmability and transferability [27, 28]. For credibility, we collected data from a large and diverse sample, using more than one data collection method to explore the same topic from multiple perspectives. We also applied more than one theoretical framework—the BeSD and the SEM—to ensure context was considered in the interpretation of the data. This study also involved investigators from various academic backgrounds, including the social sciences, public health and medicine, who conferred and agreed on coding and interpretation. Dependability was achieved through documentation of regular monthly meetings wherein progress of the project activities, preliminary data, as well as decisions made by the project team regarding issues and concerns that arose during the study period were discussed. Confirmability was established through peer debriefings to discuss findings and interpretations of the findings throughout the research process. Member checking was also accomplished through a feedback forum involving the KIs and other stakeholders in the central and regional offices of the DOH, Republic of the Philippines wherein initial findings and the researchers’ perspectives were presented. Finally, transferability was ensured by providing a detailed description of the methods and frameworks used, a description of the study sites and the research participants to enable the readers to determine applicability of the findings to contexts outside of the study.

## Results

### Characteristics of included participants

A total of 57 KIIs were conducted in the study sites, namely, Regions IV-B, V and VIII covering six provinces, and 12 municipalities. Program managers, coordinators and implementers at the national, regional, provincial and municipal levels were interviewed. From the six study sites in the three different regions, a total of 143 participants engaged in 12 FGDs (see Table 1). Most (97.9%) were female primary caregivers with a few BHWs with children aged 0–11. The age range of the participants was 19 to 76 years old, and the number of their children ranged from 1 to 10. Three quarters of participants had at least a high school education, and most were housewives (62.1%). In some of the study sites, there were participants belonging to Indigenous Peoples communities.

**Table 1:** Demographic profile of FGD participants (n = 143)

Sex	n	%
Female	140	97.9
Male	3	2.1
*Educational attainment*		
Elementary level	29	20.3
High school level	89	62.2
College level	22	15.4
No information	3	2.1
*Occupation*		
Housewife	89	62.2
Housekeeper	21	14.7
*Barangay* personnel/volunteer*	14	9.8
Farmer	6	4.2
None	8	5.6
Others**	5	3.5

*BHW, Barangay Nutrition Scholar, etc.

**Vendors, miners, encoder, seamstress

### Behavioural and social drivers of routine childhood immunization

The following sections detail the enablers and barriers to routine childhood immunization identified through both KIIs and FGDs, organized according to the domains of the BeSD framework and the SEM (See Additional file 3). Table 2 below outlines the various domains of the BeSD and levels of SEM vis a vis themes from the study.

**Table 2:** Themes under the BeSD domains and Socioecological levels

BeSD domain	Socioecological level	Themes
Thinking and feeling	Intrapersonal	Perceived benefits of the vaccines
		Fear of negative side effects of the vaccines
Social processes	Interpersonal	Influence of husbands, elders and other family members
		Influence of peers (Bakuna Champions)
	Institutional	Barangay Health Workers as influencers
		Local provision of incentives
	Community	Influence of government officials and messages
		Influence of community elders and religious leaders
	Policy	Linking immunization to the Pantawid Pampamilyang Pilipino Program
Practical issues	Intra / Interpersonal	Transportation challenges and costs
		Concern about missing work due to child’s vaccine side effects
	Institutional	Accessibility of vaccination
	Policy	Vaccine transportation and cold chain
		Availability of vaccines—stock-outs
		Movement of population (trans-out)

**Thinking and feeling.** The drivers of vaccination related to what people think and feel all operated at the interpersonal level. It was well recognized by participants of the study that vaccinations provide protection against diseases. However, the same research participants articulated fear of side effects as a major reason for not bringing their children to the health centres for vaccination. First-time mothers found this worrisome as fever was perceived as an illness rather than as a commonly occurring reaction to vaccination. Mothers would also not allow their children to get vaccinated when the children were sick with even a mild viral illness:

*“Others think that when their children are sick, giving them vaccines will make the sickness worse and that there will be complications.” (Region VIII FGD)*

**Social processes.** Influential social processes were identified at the interpersonal, institutional, community, and policy level. At the interpersonal level, in Regions V and VIII, fathers were cited as exerting a strong influence on their wives. This influence could be either an enabler, if the husband supported vaccination, or a barrier, if they did not. Participants in all study sites also mentioned the influence of elders, particularly grandmothers. Some elderly FGD participants didn’t see the value of vaccination:

*“Even without vaccination, I am still alive today.” (Region IV-B FGD).*

KIs in all regions emphasized the importance of peer-to-peer vaccine advocacy from *Bakuna Champions* campaign of the DOH in increasing vaccination uptake [29]. The campaign involves BHWs in selected municipalities and volunteers from the community:

*“One of the campaigns with good results is our Bakuna Champions. They are volunteers who are advocates of vaccines. Examples of champions are mothers and fathers.” (DPCB KII)*

At the institutional level of the health facility, BHWs were named by the participants as a vital force in shaping the perception of vaccination. BHWs are a part of the community and highly trusted. Participants felt BHWs could be easily approached for health advice:

*“Face-to-face communication done by BHWs adds a personal touch that helps convince mothers.” (Region VIII KII)*

However, interviews with the BHWs revealed that they felt they needed additional training to develop their knowledge and skills so they could carry out their tasks with confidence. In particular, the BHWs felt that they needed to be updated on facts about the vaccines for NIP and improve communication strategies so they could respond more accurately to the queries from community members, especially on concerns on side effects and how to address these. The KIs supported the sentiment of the BHWs.

At the institutional level, KIs and FGD participants identified the provision of incentives as a key social process influencing people’s motivation to get vaccinated. In some municipalities in Region VIII, children who had their routine vaccinations were given candies and hygiene kits. KIs across all regions emphasized the importance of incentives, however, KIs noted that this practice was not sustainable for the local government units and so it was not considered as a main strategy to increase vaccine uptake,

*“…when there are no incentives, people do not come.” (Region V KII)*

Influences at the community level included information from government officials and agencies. FGD participants across the different regions mentioned that the local government officials like Mayors and Barangay Chairpersons were trusted sources of information on health. They mentioned that,

*“If the information comes from the barangay [officials], I know that it’s reliable.” (Region V FGD).*

KIs in all regions also mentioned that the number of vaccinated children increased due to a recent national Supplemental Immunization Activity against measles, rubella, and polio in May 2023. However, when asked to assess an example audio visual material from this campaign, some FGD participants noted that the individuals on the image were not representative of the local Indigenous Peoples communities in their area.

In regions with Indigenous Peoples communities, the community elders/leaders were also named as strong influencers. Community members in general would follow the advice of the elders and, prior to vaccination, health workers would ask for their permission. According to one KI,

*“We invited the leaders of the [Indigenous Peoples] groups to explain to them the importance of vaccination. This helped in introducing vaccination to the IP [Indigenous Peoples] communities.” (Region IV-B KII)*

Religious leaders were also influential, though this could be either in favour of or against vaccination. In Region IV-B, a KI mentioned a religious group that was opposed to immunization. A participant in the FGD in the same region shared her religious views on vaccination and health, stating that part of the teachings in her religion was that the human body can heal itself and vaccines were not considered natural hence, these were bad for the health. However, not all members and leaders of the religious groups who oppose vaccination shared the same sentiment.

Finally, at the policy level, the *Pantawid Pamilyang Pilipino Program* (4Ps) implemented by the Department of Social Welfare and Development through its Conditional Cash Transfer was cited by KIs and FGD participants in Regions V and VIII as a vital social driver of routine immunization in children. In this program, the poorest families are provided with cash assistance if they comply with regular preventive health and nutrition services, including availing vaccines included in the NIP [30].

**Practical issues.** Individuals and families cited transportation and economic issues as barriers to vaccination. KIs in all regions mentioned that those residing in geographically isolated and disadvantaged areas, were often missed in routine immunization. According to a KI, daily wage earners were most likely to spend on daily necessities rather than on immunization, particularly when a trip to and from the health centre would cost more than a day’s toil:

*“...it depends on the budget of the household. A round trip … to the centre costs around 500 pesos (PHP 500* = *US $9).” (Region V KII)*

Another reason for missing the scheduled vaccination of the children was farming, work, and household chores. As one FGD participant said,

*“Those who missed their children's vaccination often reason out that they are busy with work …” (Region V FGD)*

When the child developed a fever due to immunization, some of the husbands had to miss work to be able to take care of their sick child, thus, resulting in loss of income for the husband. This affected how husbands felt about vaccination, and therefore how they shaped the vaccination decisions of the household.

Access to vaccination was a major challenge especially in difficult to access areas. To address this at the institutional level, KIs in all study sites shared that the RHUs employed strategies including the use of the target client list, spot-mapping target clients, tracking pregnant mothers, conducting outreach vaccination activities and house-to-house visits.

Practical issues at the policy level were identified by various KIs, such as challenges in the supply chain including vaccine procurement, transport, and cold chain equipment. In the central office, a KI attributed the inadequate supplies to budgetary limitations and failed bidding. At the regional level, a KI mentioned the concern regarding inadequate cold storage facilities resulting in the inability to accommodate the allocated vaccine supply. Transport of vaccines was also identified as a challenge to geographically isolated and disadvantaged areas with a small number of targets:

*“There are areas that are hard to reach and with only a small number of targets. Carrying the large vaccine carrier through rivers and in hikes adds additional burden.” (Region V KII).*

A KI at the municipal level mentioned that the number of vaccines they received did not match what that they requested, resulting in lack of supply. Recent intensive promotion during the Supplemental Immunization Activity motivated people to get vaccinated, but limited supply meant many people had to be turned away. As the KI explained,

*“Some people were disappointed when they were turned away because of stock outs during the MR-OPV SIA vaccinations. Some parents even got mad at the Health Care Workers (HCWs) because of false promises and wasted time.” (Region IV-B KII)*

KIs in all regions noted that another major challenge they encountered in the implementation of the NIP was the “trans-outs.” Trans-out was a term used by the KIs to refer to families that moved residence and were therefore, no longer within the area of responsibility of the RHU. They were not sure if the children were able to continue with their immunization schedules in their new location. In some areas, there were also “trans-ins” referring to children who were originally from other locations and were not considered part of the target client list of children to be vaccinated. In some RHUs, trans-ins are not included in the target client list.

## Discussion

While perceptions of benefits of vaccines motivated vaccine uptake, experience of negative side effects contributed to vaccine hesitancy among some participants. The fact that fathers lost work to care for children experiencing side effects also demonstrates the interrelated nature of personal and practical barriers. Additionally, the perception that a child who is sick cannot be vaccinated was identified as barrier to vaccination. Similar findings have been reported elsewhere such as in Iran, Malaysia and India [31–33]. This highlights the importance of trusted health workers educating parents about true contraindications to vaccination and common expected side effects and how to treat them. The development of communication skills, particularly risk communication skills, of health care providers and policy makers is vital in rebuilding trust and confidence in the government and its programs including the NIP [34, 35], particularly following the *Dengvaxia* controversy. This controversy contributed to the “crisis of confidence” that damaged not only public trust and confidence in the dengue vaccine but also the other programs of the DOH including the NIP [36].

This study highlights that other family members including fathers and grandmothers may exert a strong influence on acceptance of NIP vaccination, a finding which supports previous research. Other community influencers such as the local government leaders, religious leaders and tribal elders in the case of indigenous cultural communities, are also strong social drivers for immunization. Several participants in our study cited the *Bakuna Champions* and BHWs as role models who instil positive attitudes towards immunization. There is a growing body of evidence indicating that community-based vaccine champions can improve vaccine uptake [35, 37–40]. In the Philippines, champions have been successfully engaged in other health programs such as in the urban sanitation champions for water, sanitation and hygiene [41]. The continued training and upgrading of skills among champions and BHWs may held them in performing their roles more effectively.

KIs at all administrative levels identified a range of significant practical and logistical barriers to uptake, including vaccine availability, vaccine procurement, storage, and transport. Stock outs are a common challenge faced by many low- and middle-income countries, and they are significantly associated with reduced coverage. At the national level, KIs in our study identified challenges included financing and failed biddings. In terms of financing, the main challenge remains to be the low immunization coverage performance, which is related to underutilization of existing resources, thus there is difficulty in lobbying for further increase in the budget allocated for vaccines [42]. Vaccine stock-out in the country in recent years was affected by the transition in vaccine procurement from UNICEF to self-procurement by the DOH. There were also delays in the release of funding by the government affecting vaccine delivery [43]. Limited vaccine storage capacity was mentioned as another challenge by KIs. Ideally, procurement of vaccines is done annually with a three-month stock buffer intended for potential outbreaks and to prevent stock-outs [44]. However, because vaccine storage capacity at the Research Institute for Tropical Medicine can only accommodate 3 months of the annual supply, procurement of vaccines is done in 4 tranches [42]. Long distances, challenging road conditions, high transportation costs, and conflicting vaccination and work schedules also serve as impediments to accessibility of NIP services. Outreach services were considered as an integral part of the childhood vaccination program in other countries to align with the WHO’s Regional Framework for Reaching the Unreached Western Pacific (2020–2030) [45]. Moreover, continuing support for and recognition of the extraordinary efforts of the health centre staff in ensuring that NIP services reach the intended beneficiaries would help ensure timely delivery of vaccination services especially to disadvantaged populations [45]. Monetary and non-monetary incentives for health workers are envisioned to serve not only as recognition of their dedication to their tasks but also as motivation for them to reach program goals [46].

This study has shown that gaps in immunization records were partly due to “trans-outs”. While there were some areas where “trans-out” children received vaccination, there were areas where administering vaccines proved challenging due to the unclear immunization status of children and their non-inclusion in the initial target list of eligible children. Consequently, transfer of residence may lead to delays or missed immunization schedules. In such cases, children were reported as having incomplete immunization in their original locality. The value of updated records becomes evident in the case of “trans-outs” where the HCWs will have a basis in determining the immunization status of a patient. Public health authorities emphasized the importance of keeping accurate immunization records [47].

Effective partnerships with collaborators, whether from the government or non-government sectors, contribute to the achievement of vaccination goals. Multi component and wide-ranging strategies have been proven to address vaccine hesitancy in other countries [46, 48–50]. Global health partners of the DOH, namely, UNICEF and the WHO have also been long time partners and supporters of the NIP. In April 2023, the DOH, UNICEF and the WHO launched *Chikiting Ligtas 2023: Join the Big Catch Up, Magpabakuna para sa Healthy Pilipinas!* [51]. The WHO and UNICEF aided in procuring vaccines, deploying additional health staff for social mobilization and building cold chain capacities, among others [51]. Additionally, in the conduct of vaccine related public health initiatives particularly the World Immunization Week, the DOH urged its partners in the medical community and the private sector to intensify collaborative efforts to build strong and resilient immunization programs [52].

### Recommendations

**Build trust in the immunization program.** It is vital that trust in the program and its implementers is developed. The study shows that this may be achieved through providing culturally appropriate messaging reassuring parents about the safety and benefits of vaccines, including offering clear and practical advice for recognizing and treating common side effects. It is also important to recognize the potential influence of family members as well as community and religious leaders by engaging and empowering them to become vaccine advocates through training programs like the *Bakuna Champions* program. Moreover, it is vital to prioritize efforts to rebuild trust in government following the Dengvaxia controversy by collaborating with organizations such as the WHO, UNICEF and other expert groups to indicate a clear position on the controversy using scientific evidence.

**Build the capacities of community health personnel.** Community health personnel must be capacitated to develop the necessary skills and attitudes to carry out tasks for an effective community health program. Thus, it is necessary to train all levels of health workers including BHWs to communicate effectively about vaccination and address vaccine misconceptions. Partnerships with higher education institutions may also be explored to provide the technical support needed for the continuing capacity building of health workers in communication health leadership, and other essential skills related to vaccination. The Provincial Health Offices play a vital role in logistics support to community health centres, hence, it is necessary that provincial health office personnel are provided with technical support to ensure a dedicated and proactive health promotion unit at the provincial level that can create health promotion programs tailored to the needs of the province.

**Improve on logistics.** Monitoring of stock levels and accurate assessments of the number of eligible children must be improved to facilitate effective forecasting and timely vaccine procurement. It is likewise, vital to maximise opportunities for health promotion and education and to reduce the burden on providers and caregivers by integrating policy, advocacy, training, and service delivery of vaccines with other population-level interventions such as the Mass Drug Administration for schistosomiasis.

### Strengths and limitations

By covering three regions with low routine childhood vaccine coverage in the Philippines, this study can provide a broader view of different contexts that shape the factors affecting vaccine uptake from both the vaccinators and caregivers’ perspective. The responses obtained from the KIIs and FGDs cover all the administrative levels of the DOH from the Central Office, regional, provincial, municipal levels down to the community level. This overall design provided a general understanding of program implementation, good practices, and challenges that may contribute to enhancement of service delivery.

The study also had its limitations including the selection of the study sites. Some areas with low FIC coverage rates were not included due to logistical limitations. Recruitment of FGD participants may also be biased towards those who were vaccinated or had vaccinated children due to recruitment being conducted near health facilities. While efforts were made to enlist unvaccinated individuals and those with unvaccinated children, most of the participants were those who were vaccinated. Lastly, the timing of the study may have affected the data gathered. Field data collection began in July 2023, one month after the MR-OPV SIA campaign concluded. The campaign, which was intensive with the goal of reaching every unvaccinated child, may have shaped perspectives on the NIP in the study sites.

## Conclusions

This study found that perceptions of benefits and negative side effects remain the major intrapersonal drivers of vaccination. These perceptions were influenced by family and community relationships. There were practical issues affecting procurement, transport and cold chain facilities that impact vaccine supply. Understanding the barriers to vaccination and restoring immunization confidence and coverage in the region are critical priorities, and the findings of this study are of relevance not only in the Philippines but throughout the region. The study’s findings may be used to inform public health policies, specifically on the design and implementation of evidence-based communication strategies and health education campaigns.

## Supplementary Information

Additional file1 (PDF 33 kb)

Additional file2 (PDF 38 kb)

Additional file3 (DOCX 129 kb)

## Data Availability

The data that support the findings of this study are available on request from the corresponding author, SND. The data are not publicly available due to some pieces of information which may compromise the anonymity of the participants.

## Abbreviations

4Ps: *Pantawid* *Pamilyang Pilipino Program*
BeSD: Behavioural and social drivers of vaccination (besd)
BHW: Barangay health worker
COREQ: Consolidated criteria for reporting qualitative research
FGD: Focus group discussion
HCW: Healthcare workers
KI: Key informant
KII: Key informant interview
NIP: National immunization program
RHUs: Rural health units
SEM: Socioecological model
WHO: World Health Organization
