# Supplementary material for: Behavioural and social drivers of routine childhood immunization in selected low coverage areas in the Philippines
Source: Glob Health Res Policy. 2025 Sep 29;10:48. doi: 10.1186/s41256-025-00447-5 (PMC12477805; doi:10.1186/s41256-025-00447-5)
Supplement: Supplementary file 1 — Additional file1 (PDF 33 kb) [file 41256_2025_447_MOESM1_ESM.pdf]

Guide Questions for the Key Informant Interviews[1]

Name of Key Informant: ______________________________

Position and Office: ______________________________

Duties and Responsibilities:

1. As _(name position)_, what are your main duties?
2. What are your duties in connection with immunization against COVID-19/for children?
   - Please tell us the steps involved once the patient/child arrives at the health center for immunization.
   - What tasks/duties are performed prior to the arrival of a patient/child in the health center? When in the center? After immunization in the center?
   - Are there follow ups that you need to do? If yes, when is this done and what do you follow up on?
3. Which of the procedures would you say are the easiest to accomplish? Why do you say so? Which of the procedures do you think are most difficult to accomplish? Why?

Vaccine Uptake and Hesitancy:

4. What do you think helps in keeping families up to date with COVID-19 boosters/childhood immunization? What motivates them to return for boosters of COVID-19/childhood immunization?
5. Why do you think people hesitate to go to the health center for COVID-19 booster/childhood immunization? What is done by the center to address this?

Recommendations:

6. What do you think needs to be done to improve on the policies and programs for COVID-19 booster/childhood immunization?
7. What do you think should be done to convince people to have their COVID-19 booster shots/childhood immunization?

THANK YOU!

[1]Adapted from: WHO Social Drivers of Vaccine Uptake, May 20, 2022
