# Supplementary material for: Behavioural and social drivers of routine childhood immunization in selected low coverage areas in the Philippines
Source: Glob Health Res Policy. 2025 Sep 29;10:48. doi: 10.1186/s41256-025-00447-5 (PMC12477805; doi:10.1186/s41256-025-00447-5)
Supplement: Supplementary file 2 — Additional file2 (PDF 38 kb) [file 41256_2025_447_MOESM2_ESM.pdf]

Focus Group Discussion Guide for Heads-of-Households / Primary Caregivers on COVID-19 vaccines, National Immunization Program and IEC Material Review

Socio-demographic information
(To be collected before the start of the FGD)

Name
Age
Barangay
Educational attainment
Employment status/occupation
Number of children

FGD Guide Questions for mothers/recipients:
First, let's talk about COVID-19 vaccines for adults specifically.

- What vaccine brands for COVID-19 are you familiar with?
- How do you feel about the COVID-19 vaccine for yourself?
    - If/when someone identifies a concern, prompt for further exploration of that concern. e.g. "It sounds like you're worried about side effects. Is that something you experienced? What about everyone else, is this a concern for you? Tell me more."
- Do you feel the same about the booster as you did about the first doses? Why or why not?
- For those of you who have had the vaccine, how do you feel about that decision? How would you feel about future booster doses?
- ACCESS: Where did you learn about COVID-19 vaccines? Where/who would you go to to find answers if you had questions?
- UNDERSTANDING: How did you feel about the information you've received about COVID-19 vaccines? What do you find unclear or confusing about COVID-19 vaccines?
- APPRAISAL: How do you tell that the information you received is true?
- APPLICATION: Where did you go to get the COVID-19 vaccine? How easy would you say it was for you to get the vaccine?
- What do you think would help encourage COVID-19 vaccination for other people?

Ok, before we switch gears to talk about vaccines OTHER than for COVID-19, does anyone have anything they'd like to suggest to improve the COVID-19 vaccination program in your area?

Great. Now I'd like to talk about routine vaccines for children. So, vaccines OTHER than for COVID-19.)

- Which vaccines for children are you familiar with?
- How do you feel about these vaccines?
    - If/when someone identifies a concern, prompt for further exploration of that concern. Eg "It sounds like you're worried about side effects. Is that something your child has experienced before? What about everyone else, is this a concern for you? Tell me more"
- Are there any vaccines you didn't/won't get? Why not?
- For those of you who have vaccinated your child or for the specific vaccines you have given your child, how do you feel about that decision?
- Where do you generally get vaccines? How easy would you say it is for you to get the vaccines you want when you want them?
- What do you think would help encourage people to vaccinate their children?
- How did you learn about vaccines for children? How did you feel about the information you received?

Now I'm going to show you a few types of information and education about COVID vaccines for adults. I'd like to hear how you feel about them. I'll ask you to rate the material in a few categories, like how clear it is, and whether you like the images. I want you to give a thumbs up if you think it's good, a sideways thumb if you think it's ok but could be better, and a thumbs down if you think it's not good. Ok, does that make sense? Let's practice first - how would you rate the weather today? (check thumbs and briefly discuss to reinforce). Ok, here is the first material.

- First, has anyone ever seen this material before? Where did you see it, if so?
- In your own words, what would you say is the main message of the material? What is this material asking you to do?
- Let's rate it now - how EASY TO UNDERSTAND would you say this poster is? Thumbs up if very easy, sideways if it could be clearer, thumbs down if it's not clear at all. (record thumbs and ask a few negative raters to describe why and what would make it better)
- How does this material make you feel?
- Ok, another rating. How APPEALING and APPROPRIATE would you say the graphics/images are? Do you like them? Do they make sense? (record thumbs and ask a few negative raters to describe why and what would make it better))
- Now let's rate the WORDS/LANGUAGE USED in the material. If you think there are the right amount of words/information, thumbs up. If it's too wordy or it's

trying to express too much information, thumb sideways or down. (record thumbs and ask a few negative raters to describe why and what would make it better)

- Final rating - would you say this material is CULTURALLY APPROPRIATE? Is it offensive, does it feel foreign, or does it feel like it was made specifically for your region? (record thumbs and ask a few negative raters to describe why and what would make it better)
- What do you think would make this poster better? What other messages about vaccination do you want to be included in the poster?
