# Supplementary figures and images for: Behavioural and social drivers of routine childhood immunization in selected low coverage areas in the Philippines
Source: Glob Health Res Policy. 2025 Sep 29;10:48. doi: 10.1186/s41256-025-00447-5 (PMC12477805; doi:10.1186/s41256-025-00447-5)

**Nested Socioecological model on BeSD framework**


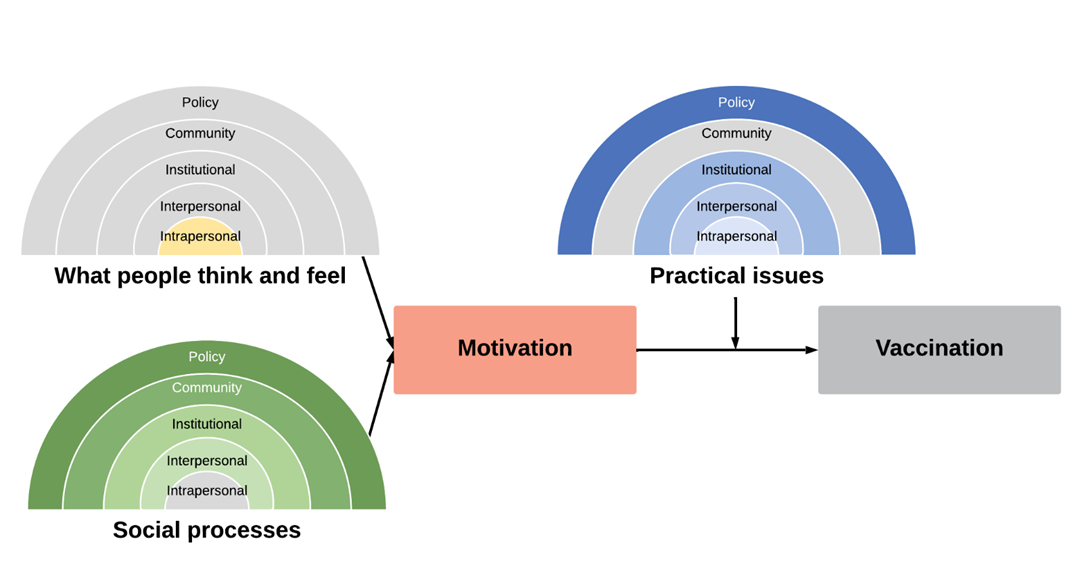

Supplement: Supplementary file 3 — Additional file3 (DOCX 129 kb) [file 41256_2025_447_MOESM3_ESM.docx]
